# Microbial responses to ocean deoxygenation: Revisiting the impacts on organic carbon cycling

**DOI:** 10.1016/j.isci.2025.112826

**Published:** 2025-06-05

**Authors:** Quanrui Chen, Kai Tang, Weidong Zhai, Zhuoyi Zhu, Jin-Yu Terence Yang, Zhili He, Meng Li, Shuh-Ji Kao, Jun Yang, Qiang Zheng, Christian Lønborg, Helmuth Thomas, Nianzhi Jiao

**Affiliations:** 1 Innovation Research Center for Carbon Neutralization, State Key Laboratory of Marine Environmental Science, Fujian Key Laboratory of Marine Carbon Sequestration, College of Ocean and Earth Sciences, Xiamen University, Xiamen 361005, China; 2 Frontier Research Center, Southern Marine Science and Engineering Guangdong Laboratory (Zhuhai), Zhuhai 519082, China; 3 School of Oceanography, Shanghai Jiao Tong University, Shanghai 200030, China; 4 Archaeal Biology Center, Shenzhen Key Laboratory of Marine Microbiome Engineering, Institute for Advanced Study, Shenzhen University, Shenzhen 518060, China; 5 State Key Laboratory of Marine Resources Utilization in South China Sea, Hainan University, Haikou 570228, China; 6 Aquatic EcoHealth Group, Fujian Key Laboratory of Watershed Ecology, Key Laboratory of Urban Environment and Health, Institute of Urban Environment, Chinese Academy of Sciences, Xiamen 361021, China; 7 Department of Ecoscience, Aarhus University, 4000 Roskilde, Denmark; 8 Institute of Carbon Cycles, Helmholtz-Zentrum Hereon, 21502 Geesthacht, Germany

**Keywords:** Earth sciences, Geomicrobiology, Biogeochemistry, Oceanography

## Abstract

Ocean deoxygenation is impacting and will also in the future impact fundamental biogeochemical cycles. This review explores the ecological functions of microbes under hypoxic and anoxic conditions, emphasizing their critical roles in carbon source-sink dynamics. We examine microbial ecosystems in both open-ocean oxygen minimum zones and China’s coastal hypoxic areas, highlighting the microbial contributions to deoxygenation driven processes. We also explore how organic carbon cycling driven by microbial heterotrophic and autotrophic metabolisms change across oxygen gradients. Furthermore, this review elucidates the interconnected cycling of carbon, nitrogen, sulfur, and phosphorus, which regulate organic matter consumption and/or storage under deoxygenation, and alters the elemental composition of organic matter. Our study highlights the importance of microbial processes in regulating carbon cycle under ocean deoxygenation, emphasizing the dual role of hypoxic zones as transient sources and long-term sinks of organic carbon. Lastly, we highlight current challenges in addressing ocean deoxygenation and provide avenues for future research.

## Graphical abstract

**Figure:**
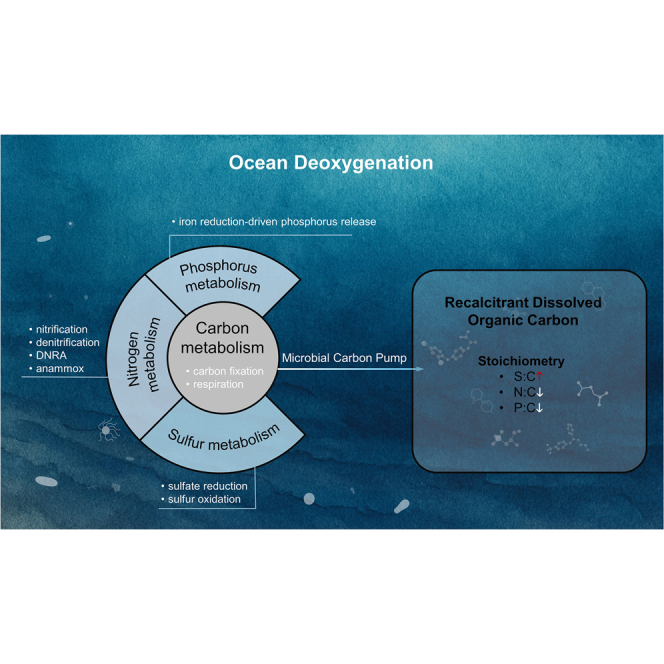

## Introduction

The ocean plays an important role in regulating global climate and biogeochemical cycles. However, marine ecosystems are currently impacted by multiple anthropogenic pressures, including acidification, warming, and deoxygenation. These stressors, both individually and in combination, pose significant challenges to fundamental ecological processes.^1^^,^^2^^,^^3^ Climate-driven changes are thereby placing critical functions such as the reproduction of larger organisms, the maintenance of biodiversity, and aquaculture production at high risk.^4^ This is primarily due to the rapid pace of change, which far exceeds the capacity of many organisms to adapt.^5^ Ocean warming is both directly (e.g., due to decreased oxygen solubility) and indirectly (e.g., due to increased respiration and stratification) reducing the ocean oxygen content (known as “ocean deoxygenation”).^6^ This impact is in coastal waters further amplified by direct human driven impacts, such as eutrophication (i.e., anthropogenic nutrient and organic matter enrichment), which increases respiration rates and results in low oxygen concentrations due to an imbalance between biological and physical sources and sinks of oxygen. Deoxygenation, impacts marine biodiversity by altering species distributions and interactions, often leading to habitat loss and restructuring of the food web, which can have cascading impacts on ecosystem functioning.^7^^,^^8^^,^^9^ Deoxygenation is typically described across a spectrum of oxygen conditions. Generally, hypoxic conditions are defined as occurring when dissolved oxygen (DO) concentrations reach levels below 60 μM.^10^ As oxygen concentrations decline further, anaerobic microbes become active (<20 μM).^11^ The water column is further classified as suboxic when DO drops below 5 μM, and anoxic condition is when oxygen reaches zero, resulting in completely anoxic or sulfidic conditions.^7^^,^^12^ These DO thresholds provide a framework for characterizing environments impacted by deoxygenation and understanding their ecological impacts.

The combined effects of ocean warming and excessive nutrient loading have already caused eutrophication-induced hypoxic conditions in over 700 coastal regions globally.^8^^,^^9^ Oceanic hypoxic environments encompass a diverse range of habitats, including mid-depth, persistent oxygen minimum zones (OMZs) in the open ocean —which are the largest regions,^13^^,^^14^^,^^15^—seasonal hypoxia in coastal waters,^16^^,^^17^^,^^18^^,^^19^ and sulfidic waters, such as those found in the Black Sea^20^^,^^21^ and Baltic Sea.^22^ While the primary drivers of these hypoxic conditions have been comprehensively reviewed,^9^^,^^16^^,^^23^^,^^24^^,^^25^ the critical role of microbial processes within these systems remains poorly understood.^26^ In particular, the coupling and interactions between microbially driven processes under deoxygenation have yet to be understood in detail. Here, a large challenge lies in establishing precise thresholds for microbial activities, as example facultative anaerobic processes can function across a wide range of DO concentrations.^11^ Furthermore, the complex interactions between heterotrophic microbial metabolism and organic carbon (OC) dynamics can further complicate how these systems respond. Although previous studies have explored the supply and degradation of OC under hypoxic conditions,^12^ the mechanisms by which microbial processes influence regional carbon source-sink dynamics remain unclear. During deoxygenation, microbial respiration can via OC degradation both release carbon dioxide (CO_2_) and exacerbate deoxygenation by consuming oxygen. Conversely, hypoxic conditions can enhance carbon sinks by slowing OC degradation, increasing sedimentary OC burial.^27^^,^^28^ These dynamic microbial processes establish a tight feedback mechanism between hypoxia intensity, duration, and OC cycling, underscoring their important role in regulating the responses in both coastal and open ocean systems.

In this review, three key aspects will be explored: (1) the ecological function of microbial communities in OMZs and under coastal deoxygenation conditions, (2) the critical processes by which microbes utilize and sequester OC during hypoxic to anoxic transitions, and (3) the interplay between microbial-driven biogeochemical cycles and the relationship between carbon sources and sinks. By addressing these aspects, we aim to provide a comprehensive understanding of how microbes influence carbon cycling in deoxygenated ocean.

## Characterization of microbial ecosystems in deoxygenated oceans

### Open ocean

OMZs occur at depths between 200 and 2000 meters in the open ocean and are characterized by a bell-shaped oxygen profile, with oxygenated waters at the surface and bottom layers, and an anoxic or hypoxic core at mid-depths.^29^ These hypoxic environments encompass a continuum, with open ocean OMZs representing the largest and most well studied regions.^6^^,^^30^ OMZs, such as those in the Eastern Tropical North and South Pacific, and the Arabian Sea, are characterized by anoxic cores that sustain anoxic nitrogen cycling processes like denitrification and anammox.^31^^,^^32^ Unlike euxinic systems such as the Black Sea, where hydrogen sulfide accumulates due to permanent stratification,^21^ OMZs rarely experience sustained sulfidic conditions, although transient sulfur plumes and cryptic sulfur cycling have been observed in some regions such as off the Namibian coast and the Eastern Tropical South Pacific.^33^ As global average sea surface temperatures will increase by approximately 3°C over the next century, the ocean oxygen consumption rate will increase 1.5-fold due to increased respiration rates.^34^ This increase in sea surface temperature, together with enhanced water stratification and the compounding effects of acidification, will further exacerbate the reduction of oxygen availability throughout the ocean.^35^ However, due to changes in ocean currents and rising temperatures —reducing oxygen solubility and increasing microbial respiration— it is projected that, by 2100, permanent ocean deoxygenation will affect 80% of the twilight zones (200–1000 m) globally.^29^^,^^36^

**Figure 1:**
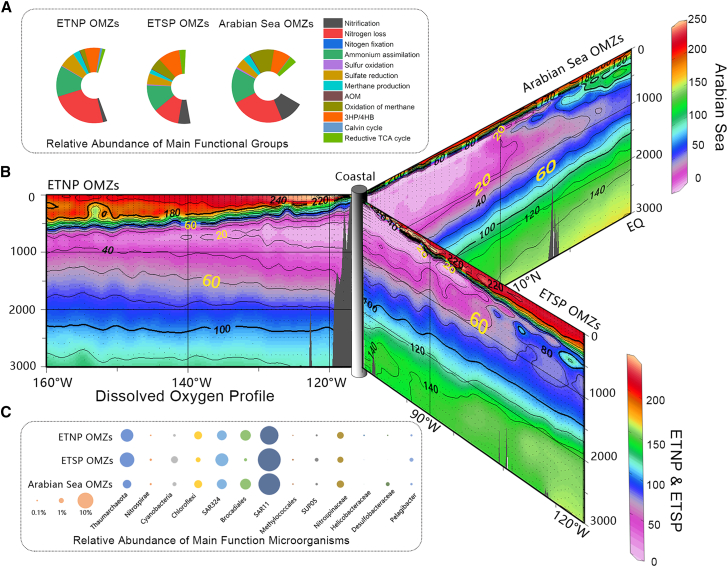
The distribution of microbial functional groups, dissolved oxygen and community composition across the Eastern Tropical North and South Pacific and Arabian Sea OMZs (A) The metagenomic features of microbial populations are presented, including the relative proportions of key functional groups of microorganisms. (B) The dissolved oxygen profile data were obtained from: www.ewoce.org/data/index.html and OMZs were defined as regions with oxygen concentrations below 60 μM. (C) The composition of microbial communities involved in carbon, nitrogen, and sulfur cycling are shown with data obtained from the Tara Ocean database (http://ocean-microbiome.embl.de/companion.html). Genomic and metagenomic data for the Eastern Tropical South Pacific OMZ were derived from stations 100, 110, and 111 (metagenomic data were only available for station 110). Data for the Eastern Tropical North Pacific OMZ were obtained from stations 137 and 138 and for the Arabian Sea OMZ from stations 037 and 038. All selected stations have DO concentrations below 5 μM.

The distribution of microorganisms in OMZs has been extensively reviewed in the past.^11^^,^^26^ In these regions stratification fosters the development of distinct microbial communities adapted to hypoxic conditions, with several taxa, including SAR11, SAR324, *Thaumarchaeota*, *Brocadiales*, and *Nitrospinaceae*, commonly found across multiple major OMZs (Figure 1). So while the microbial community structure have been reported across different OMZs, the exact composition may vary depending on local environmental conditions.^37^^,^^38^^,^^39^ The stability of microbial communities across OMZs, such as those in the Eastern Tropical North and South Pacific, indicates their shared ecological functions, particularly in supporting specialized nitrogen and carbon processes within the stable anoxic core.^40^^,^^41^^,^^42^^,^^43^^,^^44^ The resilience of these microbial assemblages to variations in oxygen concentrations suggests a stable functional framework for processes such as ammonia oxidation, sulfate reduction, and methane production, even under fluctuating environmental conditions. Within these deoxygenated zones, the dominating metabolic active microbes primarily influence the nitrogen cycle,^26^^,^^45^ but also other functional groups of microorganisms such as those involved in sulfate reduction and methane production are active. Additionally, the presence of carbon fixing microbes has been documented across three major OMZs (Eastern Tropical North and South Pacific and Arabian Sea).^43^^,^^44^^,^^46^

### Coastal environments

**Figure 2:**
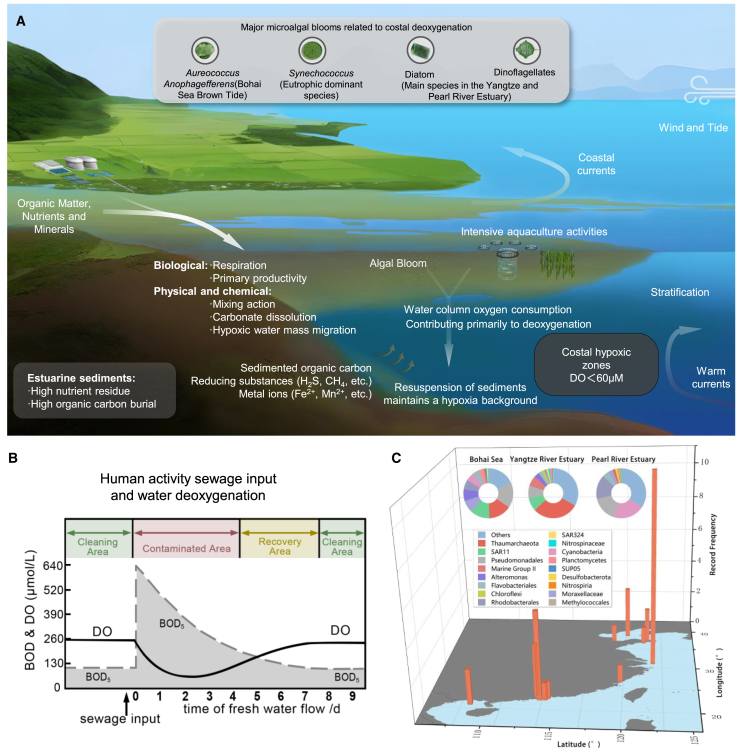
Factors influencing deoxygenation in coastal waters and records of seasonal hypoxia along the Chinese coast (A) Outline of the key factors that impact nearshore deoxygenation and possible biogeochemical feedbacks. (B) Schematic graph showing BOD responses along an estuary impacted by human sewage inputs. When receiving sewage inputs the BOD increases sharply, leading to rapid depletion of oxygen however as the water flows further offshore it exchanges with other water masses which replenishes the oxygen as shown by the black line. (C) Characteristics of different major hypoxic zones along the Chinese coast. The pie charts show the composition of the microbial community in the Pearl River Estuary,^48^ Bohai Sea^49^ and the Yangtze River Estuary (data obtained from NODE database: OER287739, https://www.biosino.org/node/). For this graph representative sequences and operational taxonomic units were reconstructed based on the sequence information provided in the respective studies. The orange bars indicate the recorded frequency of deoxygenation events (defined as oxygen concentrations <60 μM) in the corresponding regions (or stations).

Along coastlines, deoxygenation is primarily driven by (1) the degradation of OC from various sources, including sewage, agricultural runoff, and other nutrient-rich inputs; (2) increased nutrient influx, often associated with upwelling events (in which deep, nutrient-rich waters rise to the surface), that enhance plankton production and subsequent sedimentation, leading to deoxygenation in shelf sediments^47^; and (3) the advection of warm offshore water, as well as freshwater input causing salinity changes, both of which contribute to water stratification and reduce the transfer of oxygen to bottom waters (Figure 2A).^23^ When urban sewage is transported to coastal waters it results in a high biological oxygen demand (BOD) which will quickly decrease the DO concentrations (Figure 2B). This leads to deoxygenated water masses being transported downstream by rivers.^50^ Often the increased phytoplankton production of organic matter due to eutrophication can also lead to seasonal hypoxic conditions in coastal regions.^51^^,^^52^ In China, commonly, these phytoplankton communities are dominated by diatoms, dinoflagellates (primarily referring to the photosynthetic members of *Dinophyceae*), *Synechococcus*,^51^^,^^52^^,^^53^ and sometimes blooms of *Aureococcus anophagefferens*.^54^^,^^55^ As such, deoxygenation in coastal regions commonly is influenced by a wider range of factors than in OMZs.

In contrast to OMZs, coastal hypoxic environments exhibit greater variability; they are driven by local factors such as nutrient availability, sewage input, and aquaculture activities, which lead to diverse microbial communities which can change activity and composition over relatively short time scales (days). Along the Chinese coastline seasonal deoxygenation frequently occurs in various regions, particularly in the Yangtze River Estuary, Pearl River Delta, and Bohai Sea. Microbial communities in these areas have been shown to distinct compared to oxygenated environments.^56^^,^^57^ While *Thaumarchaeota* and SAR11 dominate in most hypoxic zones, near-shore deoxygenated environments are characterized by a higher abundance of opportunistic microbes, reflecting distinct community structures.^48^^,^^49^^,^^58^ For example, taxa such as the highly active *Flavobacteriales* and *Alteromonadales* groups, which are known for their ability to efficiently degrade OC, are frequently observed in high abundance in coastal hypoxic regions^59^^,^^60^^,^^61^ (Figure 2C).

In China, extensive nearshore aquaculture also contributes to deoxygenation (Table 1).^80^^,^^81^ Especially in areas of low water circulation, the excessive addition of OC and higher temperatures increase heterotrophic microbial respiration leading to both hypoxia and lower pH (i.e., acidification) in bottom waters. This combined decline in oxygen and pH, has been demonstrated in Chinese coastal waters with observations in the Bohai Sea’s seasonal hypoxic zones showing a 60%–100% increase in the bottom water H^+^ concentrations within a two month period. This corresponds to a similar change in pH levels as is projected for ocean surface waters over the next 50–100 years^83^ Recent observations also suggest that periods of intensified hypoxia in aquaculture systems coincide with elevated dissolved organic carbon (DOC) concentrations, reflecting enhanced *in situ* production of DOC and its potential role in sustaining oxygen consumption over extended periods.^80^ Additionally microbial processes in these deoxygenated waters also generate greenhouse gases, including N_2_O and CH_4_.^84^^,^^85^ These gases are partially transported to the upper ocean or the atmosphere, potentially further exacerbating global warming.

**Table 1:** Record of deoxygenated regions along the Chinese coast due to eutrophication, domestic sewage water, and aquaculture

Deoxygenation cause	Affected area	Minimum recorded DO(μM)-(year)	References
Domestic sewage water	Pearl River Estuary -Upstream	30 – (2001) 12 – (2004) 2 – (2008)	Zhai et al.^62^; Dai et al.^63^; He et al.^64^
	Xiaoqing River Estuary - Upstream	3.5^a^ – (2002)	Meng et al.^65^
	"Deep Water Bay" in Shenzhen	96^a^ – (1986–2006)	Xu et al.^66^
Eutrophication	Yangtze River Estuary	3.3 – (2016) 49 – (2018)	Zhu et al.^67^; Sun et al.^68^; Guo et al.^69^
	Pearl River Estuary	7.0 – (2014) <20 – (2010) 42 – (2017)	Su et al.^70^; Qian et al.^71^; Zhao et al.^72^
	Bohai Sea - central part	80.0 – (2014) 66.0 – (2015) 69 – (2020)	Zhai et al.^73^; Zhao et al.^74^; Chen et al.^75^
	The Mirs Bay	<32.0^a^ (1998–2010)	Li et al.^76^
	Beibu Gulf in Guangxi	117.76^a^ – (2011) 63.3^a^ – (2016)	Ma et al.^77^; Yuan et al.^78^
	Rushan Bay in Shandong	102.7^a^ – (2009)	Ran et al.^79^
Aquaculture	Muping marine aquaculture area in Yantai	49.9^a^ – (2016) 31.8 – (2020)	Zhang et al.^80^; Yang et al.^81^
	Sansha Bay aquaculture area in Fujian	135^a^ – (2011)	Wang et al.^82^

DO, Dissolved oxygen.

a Referencing the original text, DO is calculated in mg/L. For the table, the unit conversion to μM uses formula 1mg/L = 32 μM.

Additionally, coastal morphology and physical water movement also influences water column oxygen dynamics by impacting water exchange which has been shown to promote hypoxia in systems such as the Baltic Sea,^86^ Black Sea,^87^ and the Santa Barbara Basin in the United States,^88^ which contrasts with the Pearl and Yangtze River Estuaries where deoxygenation is primarily controlled by terrestrial inputs and hydrodynamics.

## Microbial organic carbon cycling under ocean deoxygenation

Deoxygenation affects vast regions of the global ocean, consequently altering biogeochemical cycles and energy conversion.^9^ In these regions, the lower presence and thereby activity of larger organisms make these microbial comminated systems. The microbial communities are here serving dual roles, acting as both contributors and degraders of OC through processes such as respiration, fermentation, and anaerobic oxidation.^12^ Simultaneously, dark carbon fixation, autotrophic metabolic processes, and the microbial carbon pump —which transforms DOC from labile to more recalcitrant forms through microbial activity^89^— contribute to carbon storage.^26^^,^^90^ Based on the above, this section will explore the bioavailability of OC, microbial carbon fixation capabilities, and organic matter sulfurization processes, while examining their influence on carbon cycling under hypoxic conditions.

### Dynamics of organic carbon

In the marine environment, oxygen consumption is intricately linked to the supply and degradation of OC by heterotrophic microbes. Heterotrophic bacteria account for approximately 12–59% of the total respiration in the open ocean.^91^ This oxygen consumption can be estimated either through direct measurements of microbial respiration rates in incubations or indirectly through the apparent oxygen utilization (i.e., difference between the oxygen concentration at saturation and the actual measured concentration in the water).^92^^,^^93^ Microbial utilization of OC in the ocean primarily involves the rapid degradation of labile particulate organic carbon (POC) and DOC in the upper ocean. Combined labile DOC (LDOC) and suspended POC can account for over 30% of the apparent oxygen utilization in the ocean.^94^ As ocean depth increases, the rate of OC consumption declines together with the bioavailability of the organic compounds, making DO a poor indicator of OC consumption in deeper ocean regions.^95^ Heterotrophic bacteria degrade LDOC within days to weeks,^96^ however, the relationship between heterotrophic bacteria and recalcitrant DOC (RDOC) —defined as DOC that is resistant to microbial utilization and can persist in the ocean for thousands of years^97^— in deoxygenated environments remains poorly understood.

In the Yangtze and Pearl River estuaries, deoxygenation is primarily sustained by POC derived from phytoplankton-based production, accounting for around 60–70% of total oxygen consumption.^53^^,^^70^^,^^98^^,^^99^ Contrary to the Peruvian upwelling system, studies show that DOC and its labile components, such as combined carbohydrates and hydrolysable amino acids, contribute up to 38% of the oxygen consumption in the upper layer, despite representing only a small fraction of the total DOC pool (1–25% for combined carbohydrates and 2–4% for hydrolysable amino acids).^100^ The remaining LDOC, not accounted for in these fractions, likely includes other bioavailable compounds, such as glycolipids, glycoproteins, and phytoplankton-derived organic matter. These findings underscore the differing roles of POC and DOC in oxygen consumption across systems: POC dominates in estuarine environments like the Yangtze and Pearl River estuaries, while DOC plays a larger role in the Peruvian OMZ. This contrast highlights the distinct carbon cycling dynamics of upwelling systems, driven by physical mixing and DOC degradation, compared to estuarine systems, where sedimentation processes prevail. Such differences should be considered the varying contributions of DOC and POC to oxygen consumption across different systems emphasize the importance of considering these differences when studying and managing hypoxic events. However, evaluating OC degradation and oxygen consumption in OMZs or anoxic environments remains a challenge. Despite recent advances in high-sensitivity DO detection,^101^ accurately quantifying trace-level consumption remains difficult.

In regions undergoing deoxygenation, the rapid degradation of OC generates substantial amounts of CO_2_, potentially contributing to localized acidification.^102^ As oxygen becomes increasingly limited, however, oxic degradation processes give way to anoxic pathways.^12^ These anoxic processes are generally less efficient in degrading OC compared to oxygenated conditions, leading to lower overall degradation rate in anoxic water columns.^103^ This transition from oxygenated to anoxic processes not only alters the rates of carbon cycling but also impacts the balance between carbon sources and sinks, particularly in anoxic systems.

### Microbial heterotrophic degradation of organic carbon

Under both aerobic and anaerobic conditions, heterotrophic bacteria communities utilize a variety of OC compounds to meet their energy and nutrient demands. Aerobic bacteria, such as *Pseudomonas* and *Sphingomonas*, thrive in oxygen-rich environments but exhibit limited growth and activity under deoxygenated conditions, resulting in slower DOC degradation.^103^ Anoxic microbial OC degradation is generally slower —by up to a factor of ten— than aerobic degradation.^104^ For instance, studies have demonstrated that under hypoxic conditions, the rate of OC degradation can decrease by more than 3-fold compared to those in aerobic environments.^20^ Additionally, when enzymes with high affinity are present, close attention must be paid, as their affinity properties may change under hypoxic conditions.^105^

However, in anoxic environments, specialized microbial communities adapt to efficiently degrade OC with groups such as SAR406, SAR202, ACD39, and PAUC34 being able to efficiently degrade complex organic compounds.^106^ When oxygen concentrations are low, facultative anaerobes or anaerobic microorganisms utilize alternative electron acceptors, such as nitrate, manganese (II) ions, ferric (III) ions, and sulfate to degrade OC.^107^ While research on sulfate-reducing bacteria (SRB) in marine systems has primarily focused on sediments, a study in the southern Baltic Sea reveals that they can contribute up to 74% of the sedimentary OC degradation.^108^ Recent ecological models and metabolic activity studies have further highlighted the widespread presence of SRB on POC and the occurrence of sulfate reduction in such settings.^109^^,^^110^^,^^111^

In deoxygenated seawater, POC drives high sulfate reduction rates, indicating strong SRB activity, complex OC compounds, such as volatile fatty acids, hydrocarbons, amino acids, polysaccharides, and aromatic compounds,^112^ to support their growth.^113^ Due to differences in the bioavailability and abundance of various compounds, particles rich in fresh organic matter are more readily remineralized. However, some OC compounds, such as acetate or short-chain fatty acids, are not degraded fully but are instead used by anaerobic microorganisms as fermentation substrates,.^114^ Therefore, accurately assessing these degradation patterns are crucial for precisely evaluating OC degradation and burial.

### The microbial carbon pump

Marine microbial communities convert a portion of the labile components into DOC through the microbial carbon pump.^89^ Anoxia can lead to changes in the DOC composition by promoting the accumulation of sulfurized compounds, carboxyl-rich alicyclic molecules (CRAM) and highly unsaturated molecules.^21^^,^^115^ These changes are mediated by redox shifts, which changes the microbial community degrading the OC and can promote sulfur incorporation into polysaccharides and lipids thereby enhancing RDOC production.^116^ The processes contributing to the persistence in the environment are complex, particularly regarding the role of abiotic sulfurization in stabilizing DOC. While it is primarily derived from biological processes, microbially produced sulfide can under anoxic conditions directly react with OC, and thereby potentially lower its bioavailability (discussed further in Section sulfur cycling and organic carbon preservation). The importance of the microbial carbon pump dependents on the rate at which microbes degrade or convert OC. Under hypoxic conditions, the activity of some heterotrophic microbial groups is limited, which in turn affects the conversion of labile compounds into recalcitrant compounds.^117^ Deoxygenation influences substrate availability, microbial community composition, and their metabolic products, leading to shifts in the process rates.^118^^,^^119^^,^^120^^,^^121^ For example, a decline in oxygen concentrations has been shown to reduce LDOC degradation rates,^103^ while also reducing the release of RDOC. Although these findings primarily focus on heterotrophic bacteria, the role of anaerobic microbes in the water column and sediments, as well as their contributions to RDOC production, deserve further investigation. To advance our understanding of these processes, it would be beneficial to include available data in a global coastal DOM database for comprehensive discussion and analysis,^122^ which could reveal broader patterns and variations in microbial contributions to OC in relation to oxygen availability.

Building upon this, the dynamic interplay between hypoxia and OC cycling reveals a series of feedback mechanisms that could regulate carbon dynamics in marine ecosystems. Hypoxic zones depend on their stability, and environmental conditions act as both OC sources and long-term sinks. In seasonal and unstable hypoxic zones, influenced by e.g., climate change and eutrophication, OC can be rapidly degraded.^123^ In contrast, stable, anoxic environments, such as the Black Sea, have overall lower degradation rates and enhanced OC storage through processes such as sulfurization.^20^ These different roles of hypoxic and anoxic zones underscore the complex feedback between microbes and OC cycling.

### Dark carbon fixation

In deoxygenated waters, dark carbon fixation (i.e., chemoautotrophs conversion of inorganic to OC) plays a critical role, especially through chemolithoautotrophic processes mediated by specific microbial taxa. In these aphotic waters, ammonia-oxidizing archaea (AOA) such as *Nitrosopumilus* and nitrite-oxidizing bacteria (NOB) such as *Nitrospinae*, utilize ammonia and nitrite, respectively, as electron donors to fuel their carbon fixation.^124^^,^^125^ Global estimates of CO_2_ fixation rates in the dark ocean range from 1.2 to 11.0 Pg C y^−1^, which accounts for between 5% and 22% of the total marine primary production, with nitrifiers (includes AOA and NOB etc.) contributing up to ∼8.8% of the carbon fixation, as observed at Station ALOHA.^126^ This highlights the significant role of AOA and NOB in carbon fixation in dark, deoxygenated waters and underscores the importance of the 3-hydroxypropionate-4-hydroxybutyrate (3-HP/4-HB) cycle and reductive tricarboxylic acid cycle (rTCA), respectively.^49^

However, in core regions of OMZs, the near absence of oxygen may limit carbon fixation, via the 3-HP/4-HB cycle, as it is less energy-efficient than anaerobic pathways such as the rTCA or reductive acetyl-CoA pathway (Wood-Ljungdahl pathway: WL) (Table 2). Based on multiple measurements of nitrification rates, NOB are likely to play a more important role in carbon fixation compared to ammonia-oxidizing microbes.^136^ Experiments have shown that *Nitrospinae* contribute 14%–35% of inorganic carbon fixation in the mesopelagic zone, surpassing the traditionally dominant *Thaumarchaeota* in deep-ocean carbon fixation.^137^

**Table 2:** Overview of carbon fixation mechanisms in chemolithoautotrophic processes within OMZs

Pathway	Oxygen Requirement	Key Carbon Fixation Enzymes	Relative ATP Cost (to CBB)	Major Chemolithoautotrophic Pathways in OMZs	References
WL Pathway	Strictly anaerobic	FDH, CODH/ACS, FTHFS, MTHFC	0.167	Anammox/Sulfur oxidation/Denitrification/Sulfate reduction	Ljungdahl^127^; Ragsdale^128^
rTCA	Facultative anaerobic	OGS, IDH, ACL, FR	0.333	Nitrite oxidation/Sulfur oxidation	Kim et al.^129^; Evans et al.^130^
3HP/4HB cycle	Microaerobic	ACC, PCC, SCR, 4-HBD	0.667	Ammonium oxidation/Sulfur oxidation	Berg et al.^131^
3HP cycle	Microaerobic	ACC, PCC, MCR, HPCS	0.557	Sulfur oxidation/Denitrification	Herter et al.^132^; Strauss et al.^133^
CBB	Aerobic	RuBisCO, PRK	1	Ammonium oxidation/Nitrite oxidation	Bar-Even et al.^134^; Liang et al.^135^

FDH, Formate Dehydrogenase; CODH/ACS, Carbon Monoxide Dehydrogenase/Acetyl-CoA Synthase; FTHFS, Formyl-Tetrahydrofolate Synthetase; MTHFC, Methenyl-Tetrahydrofolate Cyclohydrolase; OGS, 2-Oxoglutarate Synthase; IDH, Isocitrate Dehydrogenase; ACL, ATP-Citrate Lyase; FR, Fumarate Reductase; ACC, Acetyl-CoA Carboxylase; PCC, Propionyl-CoA Carboxylase; SCR, Succinyl-CoA Reductase; 4-HBD, 4-Hydroxybutyryl-CoA Dehydratase; MCR, Malonyl-CoA Reductase; HPCS, Hydroxypropionyl-CoA Synthetase; RuBisCO, Ribulose-1,5-bisphosphate carboxylase/oxygenase; PRK, Phosphoribulokinase.

Furthermore, genes associated with the rTCA cycle reductive tricarboxylic acid cycle (rTCA) have been identified in members of *Nitrospinae* within OMZs. The rTCA cycle employed by *Nitrospinae* is considerably more energy-efficient than the 3-HP/4-HB cycle used by the ammonia-oxidizing microbe *Thaumarchaeota*, resulting in 2 compared to 6–9 ATP molecules.^137^^,^^138^^,^^139^ This efficiency is also high when compared to oxygen-tolerant cycles such as the Calvin-Benson-Bassham (CBB) cycle, which consumes 3 ATP molecules per carbon fixed under aerobic conditions. Anaerobic autotrophs in OMZs also utilize other oxygen-sensitive pathways, such as the WL and the 3HP/4HB, which demand only 0.5–2 ATP molecules per fixed carbon molecule (CO_2_ or HCO_3_^−^), making them energetically advantageous under anoxic conditions (Table 2).^140^ This efficiency gives AOA and NOB a competitive advantage in OMZs, where the availability of oxygen is minimal or absent.

Previous work has also demonstrated that dark carbon fixation is closely associated with sulfide-rich environments, such as those found in the Peruvian upwelling zone. In these regions, carbon fixation rates can reach 900–1400 nM C d^−1^, which is approximately three times lower than global average phytoplankton carbon fixation rates.^141^ These rates are primarily attributed to sulfur-oxidizing bacteria (SOB),^142^ such as SUP05 and specific sulfur-oxidizing *Epsilonproteobacteria,* which account for 11–51% of the total dark carbon fixation.^143^^,^^144^ When sulfide concentration exceeds 20 μM, SOB such as *Campylobacterota*, which exhibit high carbon fixation rates,^144^ begin to dominate. These bacteria fix inorganic carbon through the rTCA cycle, with reported fixation rates being as high as 2500 nM C d^−1^ in some anoxic basins.^145^^,^^146^ This large variation in carbon fixation rates among SOB groups underscores the important impact of both community composition and environmental factors in controlling these rates.

## Linking cycling of nitrogen, sulfur, and phosphorus with carbon

**Figure 3:**
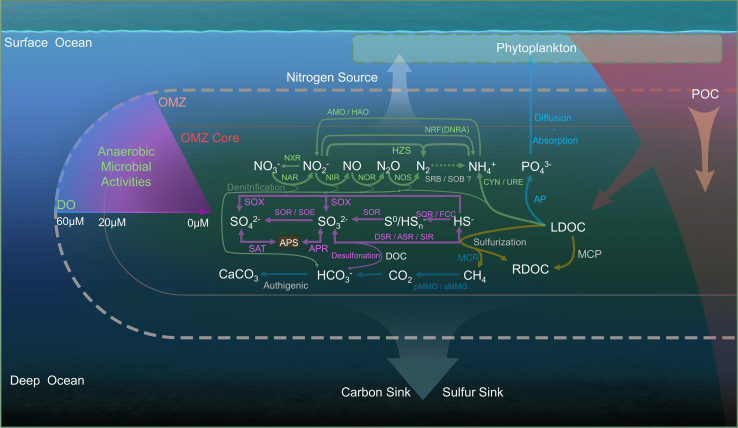
The microbially-driven cycling of carbon, nitrogen, sulfur, and phosphorus in hypoxic environments The green arrows represent microbial nitrogen transformation processes, the purple arrows represent sulfur processes, and the brown arrow represents the organic-inorganic carbon processes. Additionally, the figure highlights the regulatory enzymes, potentially contributing microbial species, and the main metabolic processes that occur under deoxygenation. SR, sulfate reduction; AOM, anaerobic oxidation of methane; NXR, Nitrite Oxidoreductase; NRF, Cytochrome Nitrite Reductase; NAR, Nitrate Reductase; NIR, Nitrite Reductase; NOR, Nitric Oxide Reductase; NOS, Nitrous Oxide Reductase; AMO, Ammonia Monooxygenase; HAO, Hydroxylamine Oxidoreductase; HZS, Hydrazine Synthase; CYN, Cyanase; URE, Urease; SOX, Sulfur Oxidation Complex; SQR, Sulfide Quinone Reductase; FCC, Flavocytochrome Sulfide Dehydrogenase; SAT, Sulfate Adenylyltransferase; APR, Adenosine 5′-Phosphosulfate Reductase; DSR, Dissimilatory Sulfite Reductase; ASR, Assimilatory Sulfite Reductase; SIR, Sulfite Reductase; SOR, Sulfite Oxidoreductase; SOE, Sulfite Oxidizing Enzyme; MCR, Methyl-Coenzyme Reductase; pMMO, Particulate Methane Monooxygenase; sMMO, Soluble Methane Monooxygenase; AP, Alkaline Phosphatase.

Despite multiple observational and modeling studies showing clear impacts of microbial metabolisms on ocean deoxygenation,^29^^,^^36^^,^^147^ direct coupling of the nitrogen,^45^ sulfur,^148^ and phosphorus^149^ cycles to this deoxygenation remains elusive. Based on our current knowledge of microbial processes in deoxygenated water, this section will provide an in-depth discussion of key studies on these elemental cycling processes and their impact on the dynamic changes of OC components (Figure 3).

The deoxygenation of microbially driven nitrogen,^45^ sulfur,^148^ and phosphorus^149^ elemental processes has been reported individually and in detail. Hypoxia alters microbial nitrogen cycling. While hypoxia promotes processes such as denitrification and anammox, these processes occur alongside dissimilatory nitrate reduction to ammonium (DNRA), maintaining nitrogen cycling under deoxygenated conditions.^41^ Concurrently, sulfate reduction generates sulfide.^150^ These denitrification and sulfate reduction processes can use OC as an electron donor to release protons or acids into the environment, impacting the marine carbon cycle (Figure 3).^151^^,^^152^ Based on current understanding of microbial ecological processes in deoxygenated water, this section will provide an in-depth discussion of key studies on nitrogen, sulfur, and phosphorus elemental cycling processes driven by microorganisms and their impact on the dynamic changes of OC components.

### Coupled nitrogen and carbon processes

In deoxygenated waters, heterotrophic bacteria can express transport proteins, such as ATP-binding cassette (ABC) and TonB-dependent transporters, which are widely distributed among Gram-negative bacteria and mediate the uptake of compounds containing both carbon and nitrogen such as amino acids and peptides.^153^ This uptake supports both AOA and NOB which can sustain nitrification under hypoxic conditions.^153^ In contrast, one study has suggested that some AOAs can release LDOC, mainly dissolved organic nitrogen, possibly as a by-product of their metabolism or as a strategy to shape surrounding microbial communities which could support heterotrophic bacterial growth.^154^

Under oxygenated conditions, ammonium released through the degradation of nitrogen-rich organic matter, such as amino acids, can be utilized by ammonia-oxidizing microorganisms including archaea. This process links heterotrophic OC degradation with autotrophic nitrification.^45^^,^^155^ AOA are the main chemolithoautotrophic microorganisms involved in nitrification and they belong to the phylum *Thaumarchaeota* which are prevalent in coastal seasonal hypoxic zones.^46^ During hypoxic events, these AOA show increased transcriptional activity and elevated expression of key enzymes (e.g., glutamine synthetase and ammonia monooxygenase) involved in organic and inorganic nitrogen metabolism.^49^ Certain AOA strains, such as the *Nitrosopumilus maritimus* strain SCM1, are capable of maintaining high ammonia oxidation activity in hypoxic environments.^156^ Under hypoxic conditions, *Nitrosopumilus maritimus* SCM1 can also maintain high ammonia oxidation activity while producing increased amounts of nitrous oxide (N_2_O), potentially enhancing greenhouse gas emissions, which thereby weakens the positive carbon sink effect in certain marine areas.

In OMZs, nitrite oxidation occurs across the entire redox gradient, including within the oxygen-depleted core. The rate of nitrite oxidation in this zone can exceed (up to 50 times) that of ammonia oxidation, with these processes being active at very low oxygen concentrations (<1 μM).^157^^,^^158^

When oxygen is absent, microbes first use NO_3_^−^ as an alternative electron acceptor. Numerous studies have investigated the role of denitrification, anaerobic ammonium oxidation, and DNRA.^31^^,^^159^^,^^160^ These processes affect the microbial turnover of OC by affecting the redox status and energy availability. In these hypoxic environments, active denitrification can produce large amounts of N_2_O.^161^ In suboxic zones, such as the Eastern Tropical North Pacific, incomplete denitrification produces N_2_O, while anammox converts ammonium and nitrite into N_2_. A recent study has revealed that N_2_O cycling in these regions is tightly coupled to OC and oxygen availability.^84^ Other studies have also shown that denitrification occurring in large particles can release N_2_O in eutrophic, high-turbidity estuaries susceptible to seasonal hypoxia.^159^^,^^162^ Such active denitrifies can regulate nitrogen turnover, which not only reduces the seawater NO_3_^−^ content but also increases N_2_O greenhouse gas emissions.^163^ Simultaneous with denitrification, DNRA can also contribute to nitrogen removal by reducing nitrate to ammonium using different electron donors, such as OC or sulfide.^164^ DNRA can compete with denitrification, particularly in environments with limited nitrate supply or high OC concentrations, such as sediments in the land-sea transition zones or sulfide rich regions.^165^^,^^166^ In OMZs, the expression of the *nrfA* gene (encoding cytochrome *c* nitrite reductase, a key enzyme in the DNRA pathway) and flux measurements suggest that the contribution of DNRA to overall nitrogen fluxes may have been underestimated.^41^^,^^167^ However, the limited availability of genetic evidence and flux measurements hampers a comprehensive understanding of the ecological importance and spatial variability of DNRA, particularly in oxygen-depleted environments.^44^^,^^168^^,^^169^ Additionally unlike denitrification, DNRA does not produce N_2_O, and therefore has a lower direct impact on greenhouse gas emissions.

### Sulfur cycling and organic carbon preservation

In suboxic water columns, dissimilatory sulfate reduction is confined to localized microenvironments, such as anoxic microsites within sinking particles or microaerophilic zones,^109^ whereas sulfide oxidation occurs preferentially within the chemocline (oxycline and/or nitricline) of stratified systems such as the Black Sea.^170^^,^^171^ Microbial sulfur cycling causes a pronounced isotope fractionation, primarily favoring the lighter ^32^S isotope. Previous work has also demonstrated that microbial sulfate reduction produces sulfide significantly depleted in the ^34^S isotope.^171^ Recent enzymatic studies have revealed that this isotope effect is predominantly governed by the enzyme Adenylyl-sulfate reductase, which causes an fractionation factor of approximately 20%.^172^ When SRB oxidize OC using sulfate as an electron acceptor, complete oxidation of the substrate typically results in the production of hydrogen sulfide with a negative δ^34^S signature. However, under non-limiting conditions, there is no simple relationship between sulfate reduction rates and isotope fractionation.^173^ Earlier studies proposed that this high isotope fractionations may reflect the high metabolic capacity of SRB.^174^ Opposite to this more recent work with the sulfate reducer *Desulfovibrio* sp. show that similarly large ^34^S values can also result from a slow metabolism.^175^ These findings imply that ^34^S fractionations do not uniquely indicate high metabolic control or energy availability, but may instead reflect low-energy states in which reversibility of enzymatic steps governs the extent of isotope partitioning.

In contrast, sulfur oxidation, traditionally assumed to produce minimal isotope effects, has more recently been shown under nitrate-reducing conditions to enrich ^34^S relative to sulfide.^175^ Moreover, sulfide oxidation via the enzymes sarcosine oxidase (Sox) and Sulfide:quinone reductase (SQR) pathways yields modest negative isotope fractionation.^176^ Therefore, both the reductive and oxidative branches of the sulfur cycle can impart diagnostic isotopic signatures, which is shaped by metabolic pathway-specific isotope effects, enzymatic control points (e.g., SQR), and ecological constraints such as redox zonation and electron donor/acceptor availability.

In the metabolism of SOB, sulfide serves as an electron donor, transferring electrons via intermediates such as reduced quinones to terminal electron acceptors, typically O_2_ or NO_3_^−^. In this process, DOC is the primary OC source and it is therefore not directly involved in the electron transfer chain.^148^ This process results in the production of elemental sulfur (S^0^) and thiosulfates (S_2_O_3_^2−^), which are further utilized by microbes through defined pathways such as the dissimilatory sulfite (Dsr) and Sulfide: quinone reductase (Sox) systems, or disproportionation reactions, to synthesize and transform organic sulfur compounds.^177^^,^^178^

Experimental evidence indicates that the sulfur-containing molecular structures formed via sulfurization of OC under anoxic conditions closely resembles the composition of dissolved organic sulfur found in sediment pore waters.^179^ These sulfurized compounds can contribute to nucleophilic addition reactions, which involve the reaction of a nucleophile with an electron-deficient carbon atom, such as olefins, carboxylic acids, and other organic compounds that contain aldehydes and ketones.^180^ These reactions result in the formation of compounds such as thioethers, thioesters, and sulfates.^179^ The CRAM, with unsaturated carbon-carbon double bonds, are a representative component produced by the microbial carbon pump, and it can further be modified by e.g., bisulfide (HS^−^) to form sulfur-containing carboxyl-rich alicyclic molecules.

However, only a limited number of microbes are capable of tolerating and utilizing sulfide,^181^^,^^182^ as high concentrations are generally toxic which implies that most OC will not be immediately degraded in these environments.^183^ Concurrently, sulfites, which are intermediates products in the microbial sulfur cycle, can, through nucleophilic addition reactions with OC, produce molecules that can resist microbial and chemical degradation, thereby persisting in anoxic environments.^184^

Even though SRB utilize a relatively low-energy electron acceptor they are highly efficient in extracting energy from the environment. Sulfate reduction produces intermediates such as polysulfides, sulfites, and elemental sulfur, which are involved in OC degradation and can here be used as indicators of dynamic sulfur–OC interactions in anoxic zones.^185^ Anoxic waters harbor a diverse community of sulfur-metabolizing microbial groups however these show varying activity. One example of this is sulfate reduction rates which generally are around 2.3 times higher in the Black Sea than those in the Eastern Tropical South Pacific OMZs,^185^^,^^186^ which may be explained by the more stable and persistent euxinia (i.e., free hydrogen sulfide) in the waters of the Black Sea. Stable anoxic conditions have been found to enhance the activity of some SRB genera, especially *Desulfatiglans* and *Desulfuromonas*.^187^ Such high sulfate reduction rate can enhance OC degradation in marine sediments, resulting in the production of bicarbonate ions and an increase in alkalinity,^188^^,^^189^ thereby facilitating CO_2_ uptake and carbonate precipitation.

Sediment column records reveal large amounts of OC and pyrite in black shale layers.^110^ The Sulfur:Carbon ratio of organic matter in these layers are similar to those in modern near-anoxic oceans, suggesting that OC sulfurization in these regions could contribute to the OC storage.^110^^,^^190^ An experimental study supports this, as they show that microbial sulfate-reduction enhances OC preservation by forming sulfurized OM.^110^ Additionally, have field studies in the Black Sea shown that recently produced dissolved organic sulfur, accumulated in the water column.^21^ With intensifying ocean deoxygenation, such organic sulfur compounds may increase OC storage with some studies suggesting a 1.5–3 times increase.^191^ Sulfonates represent one form of organic sulfur which are commonly found at the interface between oxygenated and anoxic environments. The conversion of organic sulfides into sulfonates, involving electron loss or oxidation, has garnered significant scientific attention. Examples include key organic sulfur compounds such as dimethylsulfoniopropionate, 2,3-dihydroxypropane-1-sulfonate, and sulfoquinovose, which play crucial roles in the marine sulfur cycle.^150^^,^^192^^,^^193^ these compounds being typically rapidly degraded in oxygenated environments and thereby contributing to oxygen consumption. This degradation is hypothesized to occur preferentially in environments that possess elevated pH levels, with organic sulfides potentially being oxidized to sulfonates.^194^

### Phosphate release and organic phosphorus dynamics

In the ocean, phosphorus serves as a critical limiting factor for primary productivity. Most phosphorus initially enters sediments as organic detritus, and part of it precipitates *in situ* as authigenic minerals (e.g., apatite) before potential storage.^195^ In these environments, iron oxides exert a strong control on the phosphorus cycle: under oxic conditions, phosphate is adsorbed to Fe(III) oxides; under anoxic conditions microbial dissimilatory iron reduction (e.g., by *Geobacter* or *Shewanella*)^196^^,^^197^ reduces Fe(III) to Fe(II), dissolves iron oxides and thereby releases previously adsorbed phosphate into porewaters and the overlaying waters.^198^ At the same time, biologically mediated iron oxidation can counteract this release process under suboxic or oxic conditions.^199^^,^^200^ For instance, filamentous sulfur-oxidizing cable bacteria (family *Desulfobulbaceae*) perform electrogenic sulfur oxidation by connecting deeper, sulfidic sediments with surface oxic layers.^201^ All these processes influence phosphate regeneration in bottom waters, which can in turn influence oxygen consumption and OC degradation.

Beyond metal-associated processes, microbes are also active parts of the phosphorus cycle and these links are crucial for determining the impact of deoxygenation on OC sources and sinks.^202^ However, the relationship between phosphorus and microbes ocean deoxygenation has not been investigated in detail. In general, heterotrophic bacteria utilize alkaline phosphatase to hydrolyze organic phosphorus, thereby releasing inorganic phosphate to meet their nutritional needs.^203^ Some large bacteria that reside in sediments, such as *Thiomargarita namibiensis* and cable bacteria, promote the production of phosphate minerals in the environment through their own preserved polyphosphates.^201^^,^^204^ Studies have also suggested that bacteria in deoxygenated environments have a higher relative content of organic phosphorus compared to oxic environments.^205^ This difference may reflect an adaptive strategy with microbes accumulating phosphorus within their cells, releasing phosphate or forming phosphate minerals upon organic matter degradation.^201^^,^^204^ In addition with hypoxic conditions microbial degradation of organic matter leads to the preferential release of organic phosphorus into bioavailable inorganic phosphorus.^206^ Re-oxygenation or disturbance of the environment can also release phosphate-rich water which might stimulate new OC production and degradation (i.e., respiration) and delay the return of higher oxygen concentrations.

### Stoichiometry of organic matter

**Figure 4:**
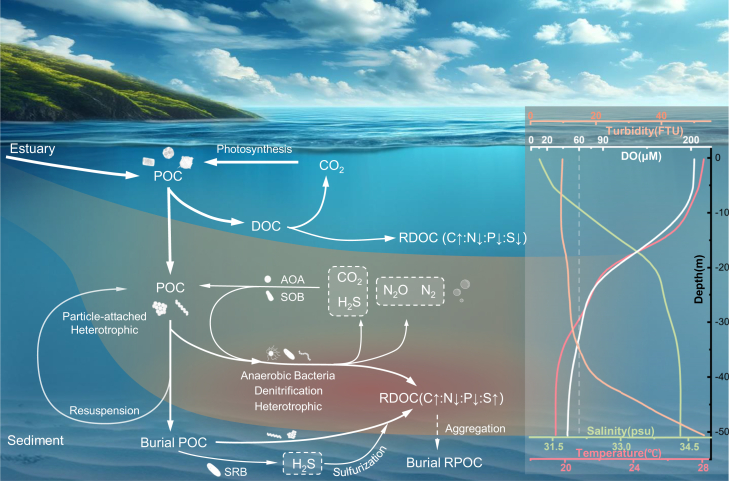
Microbial degradation and storage of OC in hypoxic coastal waters This figure illustrates seasonal hypoxic water masses, microbial metabolism in both oxygenated surface and hypoxic bottom layers predominantly driven by POC inputs from terrestrial sources and the upper ocean. Heterotrophic processes, denitrification, and the sulfate reduction in contribute to the transformation of POC,^110^^,^^159^ which can subsequently be transported offshore. Concurrently, dark carbon fixation and sulfur oxidation produce POC. In shallow nearshore regions, POC burial is furthermore influenced by resuspension, which can transport POC and sulfurized OC into bottom waters.^187^ The physical and chemical properties of a water column profile in the Yangtze River Estuary (august 2022) is also shown to the right. DOC,dissolved organic carbon; RDOC, recalcitrant dissolved organic carbon; POC,particulate organic carbon; RPOC, recalcitrant particulate organic carbon; SRB,sulfate-reducing bacteria; SOB,sulfur-oxidizing bacteria; AOA,ammonia-oxidizing archaea; DO,dissolved oxygen; C: N: P: S, Molar ratio of carbon, nitrogen, phosphorus, and sulfur.

Organic matter stoichiometry is critical for understanding potential sources and degradation pathways. Here, the Redfield ratio (marine phytoplankton Carbon:Nitrogen:Phosphorus ratio = 106:16:1) is suggestion a balance between oceanic biological productivity and the chemical composition of seawater.^207^ The microbial degradation and transition from labile to recalcitrant DOC has been shown to result in large changes in the C:N:P stoichiometry, changing from 199:20:1 to 3511:202:1.^208^ Anoxic microbial and chemical processes under anoxic conditions alter the elemental and molecular composition of organic matter, which can lead to the preservation of OC. In anoxic environments, microbial OC degradation and the prevalence of sulfurization can result in the production of organic matter rich in sulfur but deficient in nitrogen (Figure 4).^116^ Because nitrogen and phosphorus are essential yet frequently limiting nutrients, heterotrophic microorganisms preferentially hydrolyze biomolecules that contain abundant N or P—such as proteins, nucleic acids and phospholipids—to satisfy their anabolic demand for amino acids, nucleotides and membrane phospholipids,^155^^,^^209^^,^^210^ leading to the direct release of phosphate. In addition, the peptide and phospho-ester bonds in these substrates are chemically more labile than the C–C or aromatic bonds dominating N- and P-poor compounds (e.g., carbohydrates or lignin-derived aromatics).^211^^,^^212^ Microbial degradation preferentially targets organic matter that is rich in nitrogen and phosphorus,^155^^,^^209^^,^^210^ This series of processes not only reduces the nitrogen and phosphorus content in DOC, resulting in a 17.6-fold decrease in the P:C ratio, a 1.7-fold decrease in the N:C ratio, and a 3.7–6.85-fold increase in the S:C ratio,^179^^,^^208^ but also alters the bioavailability of DOC.^213^^,^^214^

Additionally, the P:C ratio of sulfidic environments are around 49 times lower than the global average for particulate organic matter and 80 times lower than that found for phytoplankton.^215^^,^^216^ Anaerobic microbial processes and chemical reactions under anoxic conditions can alter the elemental and molecular composition of organic matter. For instance, these processes can lead to sulfurization and the preservation of highly reduced organic molecules. Such transformations are evident in marine sediments which are analogous to geochemical changes observed during past Oceanic Anoxic Events, where widespread anoxia triggered large-scale sulfurization and OC storage (e.g., Mesozoic Ocean about 252 to 66 million years ago).^217^ The parallels between modern anoxic marine environments and paleoceanographic conditions underscore the role of anoxia in shaping both short-term elemental composition and long-term OC storage in the ocean.

## Summary and future directions

This review examines the impacts of ocean deoxygenation on microbial communities and OC cycling, emphasizing their potential contributions to global carbon dynamics and climate change. Ocean deoxygenation is altering the composition and functionality of microbial communities and affecting the degradation and transformation pathways of OC. A singular focus on the carbon cycle is however insufficient to fully elucidate the mechanisms taking place under deoxygenation. Microbes demonstrate remarkable metabolic flexibility, employing diverse pathways to sustain their survival and activity in deoxygenated environments. Despite considerable progress in understanding the effects of ocean deoxygenation on microbial metabolism and OC transformation, several critical issues remain unresolved. One example being the precise quantification of OC degradation rates and microbial metabolic pathways under varying oxygen conditions. Determining such complex links will require the development of advanced high-resolution measurement techniques and the integration of multi-omics approaches, including transcriptomics and metabolomics.

Future research should also prioritize understanding the interactions among microbial communities and their response mechanisms in these complex environments, especially in regions undergoing alternating long-term and seasonal hypoxia. Rising ocean temperatures and the expanding extent of deoxygenation are expected to drive changes in marine OC storage and the dynamics of carbon source-sink relationships, with clear implications for global climate. Understanding the role of ocean deoxygenation in microbial ecology and carbon cycling is therefore of critical importance for future marine management and understanding impacts of climate change. Therefore, should studies prioritize the integration of multidimensional data to investigate the feedback mechanisms linking microbial communities and carbon cycling under deoxygenated conditions. While the mechanisms of deoxygenation in open-ocean and coastal environments are well understood, challenges remain in integrating ecological processes to develop precise predictive models. Consequently, future research should address not only current environmental changes but also anticipate emerging trends, providing critical scientific insights to tackle global climate change and manage ecological systems.

## Acknowledgments

This work is supported by the National Natural Science Foundation of China project (42141003, 42188102, 92251306, and 41976042), the National Key Research and Development Program of China (2020YFA0608300), the Ministry of Science and Technology (MOST) ONCE project. During the drafting of the manuscript C.L. received funding from the Independent Research Fund Denmark grant no. 1127-00033B and the MEL senior visiting fellowship program (Xiamen University, China).

## Author contributions

N.J. and K.T. conceptualized the central idea and supervised the research. Q.C. was responsible for the comprehensive writing and editing of the manuscript, including text and figures. W.Z. and Z.Z. compiled the content on coastal deoxygenation in China. J.-Y.T.Y. and S.-J.K. provided detailed environmental characteristics of hypoxic zones in the Yangtze River estuary. Z.H., M.L., J.-Y.T.Y., and Q.Z. contributed to the revision of the sections on microbial elemental cycling under hypoxic conditions. C.L. and H.T. edited and contributed to the section on microbial OC cycling under deoxygenation. All authors participated in the interpretation of the review and contributed to the refinement of the manuscript.

## Declaration of interests

The authors declare that they have no competing interests.
